# You Know What It Is: Learning Words through Listening to Hip-Hop

**DOI:** 10.1371/journal.pone.0028248

**Published:** 2011-12-21

**Authors:** Paula Chesley

**Affiliations:** Department of Linguistics, University of Alberta, Edmonton, Alberta, Canada; Umeå University, Sweden

## Abstract

Music listeners have difficulty correctly understanding and remembering song lyrics. However, results from the present study support the hypothesis that young adults can learn African-American English (AAE) vocabulary from listening to hip-hop music. Non-African-American participants first gave free-response definitions to AAE vocabulary items, after which they answered demographic questions as well as questions addressing their social networks, their musical preferences, and their knowledge of popular culture. Results from the survey show a positive association between the number of hip-hop artists listened to and AAE comprehension vocabulary scores. Additionally, participants were more likely to know an AAE vocabulary item if the hip-hop artists they listen to use the word in their song lyrics. Together, these results suggest that young adults can acquire vocabulary through exposure to hip-hop music, a finding relevant for research on vocabulary acquisition, the construction of adolescent and adult identities, and the adoption of lexical innovations.

## Introduction

“Everything that hip-hop touches is transformed by the encounter, especially things like language… which leaves [itself] open to constant redefinition.” Jay-Z [1] (p. 80–81)

In 1979, *Rapper's Delight* by The Sugarhill Gang became the first hip-hop song to receive national radio play in the U.S. For many outside of New York City, *Rapper's Delight* was a first exposure to hip-hop. Couched in all the novelty was a non-mainstream vocabulary, much of which was African-American English (AAE; also called African-American Vernacular English or Black English). AAE words used in *Rapper's Delight*, such as *fly* (“cool/attractive”) and *bad* (“cool”), subsequently enjoyed some prominence in Mainstream American English (MAE; also called “Standard” American English) throughout the 1980s. Could it be that non-African-American speakers learned these words through listening to hip-hop songs such as *Rapper's Delight*?

Possibly. A speaker's vocabulary grows dramatically during adolescence and young adulthood [2], although more research is severely needed to investigate how this occurs. During these stages, various media forms are used for socialization purposes [3]. Perhaps it is not surprising that, given enough visual and linguistic context, speakers appear to acquire vocabulary from watching movies or television shows [4].

It is less clear, however, that vocabulary acquisition can take place through listening to music, particularly hip-hop. Even outside of hip-hop, listeners often misunderstand lyrics: they were seven times more likely to incorrectly transcribe sung words than spoken words [5]. Thus we would expect music listeners to have only a thematic understanding and memory of lyrics; previous research suggests this is the case [6], [7]. Furthermore, several barriers make it difficult to adequately understand hip-hop lyrics in particular [8], [9]:

- The lack of lyrics available in album liners, which is far more common in hip-hop than in rock and pop albums;
- The presence of background music and samples;
- The fast pace of many rappers, often too fast for comprehension (in this paper I adopt a generally accepted distinction between *rap* and *hip-hop*: *rap* consists of spoken rhymes, while *hip-hop music* constitutes the musical genre that raps often occur in [10]. In the target demographic, it is highly probable that most exposure to raps occurs through listening to hip-hop music; hence this study examines learning through *hip-hop music*, or *hip-hop* for simplicity);
- The voice quality, which can be excited, shouting, or otherwise emotionally charged;
- Unfamiliar language. Like other forms of verse, this includes atypical syntax and lexical items that better conform to verse structure. Hip-hop is also rife with double entendres and deliberately obscure language [11] (p. 73). For speakers of MAE, the prevalence of specific AAE vocabulary can make hip-hop lyrics even more difficult to understand.

These factors make for “excruciatingly difficult” conditions for lyric comprehension and transcription [9], not to mention subsequent vocabulary acquisition. A well-researched example of vocabulary acquisition under suboptimal conditions concerns hearing-impaired populations, who have lower vocabulary acquisition rates than their non-hearing-impaired counterparts [12]. Similarly, it seems challenging, perhaps especially for non-African-Americans, to acquire vocabulary as a result of listening to hip-hop.

On the other hand, adolescents and young adults listen to quantitatively more music than previous generations [3]. For example, 79% of American teens and three-quarters of 18–24 year-olds have an mp3 player [13]. Due to the ubiquity of not only mp3 players but also smartphones and internet connectivity, these populations receive increased linguistic input from popular culture icons such as music artists. From its South Bronx roots and subculture origins, hip-hop music has evolved to be fully mainstream [10]; many adolescents and young adults are regularly exposed to it. In listening to the same songs more, listeners benefit from repeated learning, enabling them to better process details. Furthermore, the availability of videos online enables immediate video viewing, which could facilitate vocabulary acquisition by offering visual context accompanying unclear lyrics whenever listeners want to watch. Websites devoted to slang, such as Urban Dictionary (http://www.urbandictionary.com/http://www.urbandictionary.com/), and to hip-hop/AAE vocabulary in particular, such as Rap Dictionary (http://www.rapdict.org/http://www.rapdict.org/), allow for explicit querying of words with unclear meanings, and Rap Genius (http://rapgenius.com/http://rapgenius.com/) aims to explicitly decode hip-hop lyrics, with or without new vocabulary.

Widespread listening to particular artists using the same words could lead to large-scale vocabulary acquisition across social groups. In fact, given the increasing prevalence of the media in young adults' lives, it is surprising that few studies examine first-language vocabulary acquisition through the media. This is particularly important research since vocabulary acquisition represents long-term learning: unlike speed, memory, and reasoning skills, vocabulary skills improve with age [14].

The context of AAE in the U.S. is ideal for testing vocabulary acquisition through listening to hip-hop music. Although AAE and MAE are mutually intelligible, AAE has regular linguistic features, including vocabulary differences, that make it a legitimate, distinct variety (or *dialect*) from MAE. Due to continued segregation patterns for African-Americans [15], [16], many MAE speakers might rarely interact with speakers of AAE. When they do, it is possible that AAE speakers do not use full-fledged AAE [17]. On the other hand, as the prestigious linguistic variety amongst hip-hop artists, AAE is often used in hip-hop lyrics [18]. Hip-hop could thus represent a primary means of exposure to AAE vocabulary for many MAE speakers.

To see if speakers might be learning AAE vocabulary from hip-hop, I studied speakers' comprehension vocabulary of AAE by asking them to give definitions for a subset of AAE lexical items that could likely occur in hip-hop songs. Due to hip-hop's genre-specific themes of violence, however, the AAE vocabulary used in hip-hop should not be considered representative of AAE vocabulary in general. The term *grip* is an example of a stimulus of item in this study, occurring in the 2003 Jay-Z song “Dirt off your shoulder” at 1∶52–1∶56:

(1) I paid a grip for the jeans, plus the slippers is clean Anecdotally, some listeners report not understanding the above lyric at all because of the fast tempo, but the visual context of the video (http://www.youtube.com/watch?v=Oz_-VaTHpc8) could help speakers interpret *a grip* to mean “a lot”. Other vocabulary in hip-hop lyrics can be more or less easy to interpret. The following study provides evidence that comprehension vocabulary of stimulus items such as *grip* stems in part from listening to hip-hop.

Because these words are not (yet) well-known by many speakers of MAE, it is likely that familiarity with these words will have some link back to the AAE speech community, either from social ties or from exposure to popular culture including hip-hop music. I hypothesized that a preference for hip-hop music would be positively associated with participants' AAE comprehension vocabulary. If however the communication channel is too noisy for vocabulary acquisition, as previous research seems to suggest, then we would expect no effect of a preference for hip-hop music on AAE comprehension vocabulary.

Several control factors were also assessed to determine whether a preference for hip-hop was not masking any underlying variables actually responsible for AAE comprehension vocabulary. A series of demographic variables such as age, sex, ethnicity, and geographic area are important for other types of language use by adolescents and young adults (see, inter alia, [19]–[22]). Second, social network variables such as strong and weak ties (roughly, close friends/family vs. acquaintances) are also important factors in the diffusion of linguistic innovations [23], [24]. Finally, general popular-culture knowledge could be responsible for knowledge of AAE vocabulary. This possibility was assessed by asking participants questions about popular culture that college-aged students could know.

## Results

**Figure 1:**
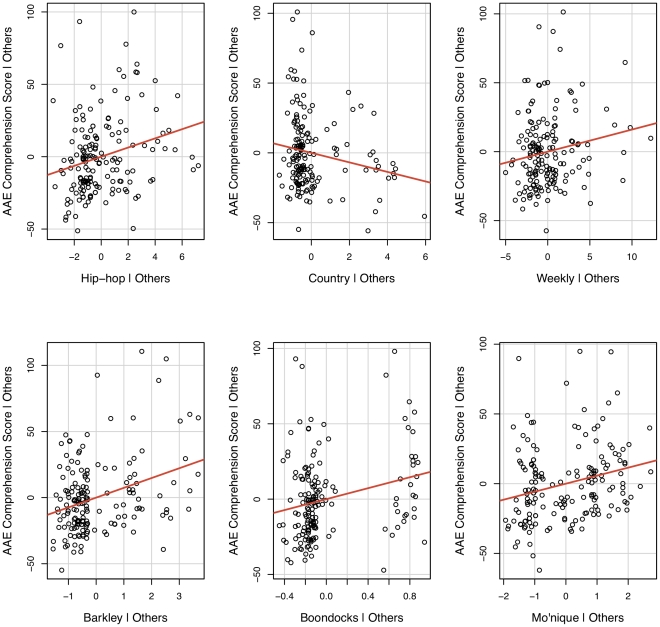
Added-variable plots of significant predictors for AAE comprehension vocabulary.

In a multiple linear regression analysis, results from the survey indicated that musical preferences, weak social ties to African-Americans, and knowledge of popular culture were significant in predicting a participant's AAE vocabulary score. Specifically, the more hip-hop artists participants listened to, the more they were familiar with the AAE vocabulary items tested. Additionally, participants' weak social ties to African-Americans and their knowledge of elements of African-American popular culture such as Charles Barkley, Mo'nique, and the comic strip and TV series *The Boondocks* were significantly associated with increased AAE vocabulary knowledge. In contrast, an increased preference for country music was negatively associated with knowledge of AAE vocabulary items. The model in Table 1 has a multiple 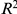 of 0.39. These results are visualized in the added-variable plots of Figure 1, which plots the partial correlations between predictor variables on the x-axes and the dependent measure on the y-axes after partialing out all other predictor effects.

**Table 1:** Model coefficients - overall vocabulary score.

	Estimate	Std. Error	t value	Pr( 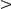 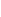 t 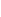 )
Intercept	78.1890	5.2616	14.8604	0.0000
Hip-hop	3.1670	0.9856	3.2133	0.0016
Country	−3.4141	1.4677	−2.3261	0.0213
Weekly	1.6055	0.7449	2.1554	0.0326
Barkley	7.4085	1.6839	4.3997	0.0000
Boondocks = yes	18.1671	5.5942	3.2475	0.0014
Mo'nique	5.7439	1.7387	3.3036	0.0012

N = 166. Dummy coding was used for the Boondocks variable, and the reference level is no knowledge of a *Boondocks* character.

Demographic variables such as sex, age, and hometown population information (city and county populations, as well as the county's African-American population) were not significant predictors, nor were the strong-tie variables to any ethnicity.

To ensure that the effects were not due to multicollinearity, I inspected pairwise correlations between predictors; correlations were modest [25] (p. 138). For example, the numbers of hip-hop and country music artists participants listened to were only weakly correlated (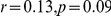). The highest correlation between predictors in the model (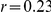, between the number of hip-hop artists listed and the number of weak ties to African-Americans), was similarly low. The variables in the model also had low correlations with those not in the model, such that the former are not masking the explanatory power of the latter. The highest such correlation was 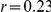, between the number of hip-hop artists a participant listed and his or her hometown county population.

To summarize, the number of hip-hop artists a participant listens to was predictive of his or her AAE comprehension vocabulary. This effect was still significant when other factors, such as participant demographics, knowledge of popular culture, and overall musical preferences, were taken into account.

### The effect of preferred artists

I next examined the lyrics from specific artists participants listen to in order to establish a firmer connection between speakers' actual linguistic input from hip-hop music and their AAE comprehension vocabulary. The use of these words by specific artists participants listed was used as an additional variable to predict participants' knowledge of the words.

**Figure 2:**
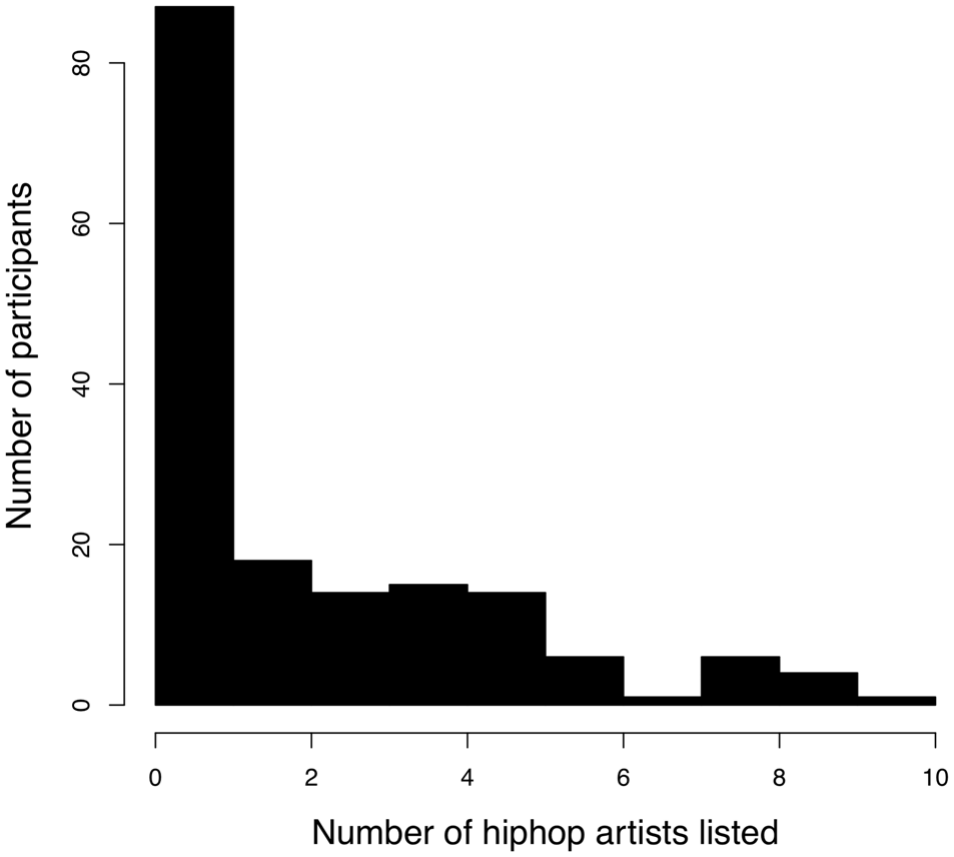
Summary of the number of participants who listed listening to hip-hop artists.

Seventy-nine of 166 participants listed listening to at least one hip-hop artist (48%; see Figure 2). I then did an automatic Google query for each hip-hop artist listed together with each word on the Urban Lyrics website (www.urbanlyrics.comwww.urbanlyrics.com). Of the 92 total hip-hop artists listed, 86 were present in the Urban Lyrics database. The lyrics each of these queries returned were manually examined to see if the usage of the word corresponded to a specific AAE meaning.

The results for each word use by individual artists were then matched to participants' preferred artists such that for each word an estimate was made for the exposure of each participant to the word through proactive listening to hip-hop. For a given word, the number of artists a participant listens to that use that word was used to predict the participant's familiarity with that word. The number of different contexts a word occurs in appears to be a better predictor of lexical processing (naming and lexical decision latencies) and later use in a speech community than overall frequency counts [26]–[28]; the same is likely true of comprehension vocabulary. Hence the number of artists using a word was used as a predictor instead of word frequency counts (see supporting information S1 for results with number of songs as a predictor instead).

This new variable was added to a mixed-effects model that included as fixed effects all significant predictors from the previous study. Mixed-effects models are ideal for this analysis because they allow for distinct fits for each participant-item pairing. The crossed random effects of Participant and Word were also included in the model.

Forty-two of the 64 vocabulary items initially tested were detected in at least one hip-hop artist's lyrics with the intended meaning. In this analysis, the number of weak ties to African-Americans and knowledge of Charles Barkley did not reach significance. However, general hip-hop and country music preferences and knowledge of Mo'nique and *The Boondocks* were still significant, with similar estimates. In addition, the number of participants' preferred artists using the word was positively associated with increased African-American English comprehension vocabulary scores (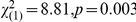).

A possible confound in this study is the level of entrenchment of a word in the AAE or MAE speech communities. Speakers might be familiar with a word in a hip-hop song because a high degree of use by hip-hop artists could be reflective of the fact that it is already in use in the MAE speech community, for example. The more a word is entrenched in a listener's speech community, the more likely it is that exposure and learning would come from a source other than hip-hop. For example, a participant might not listen to Lil Wayne, who uses *fetti* (“money”) more often than other artists in the present data. However, she could learn *fetti* in talking to friends who do.

We can approximate a words' level of entrenchment in the AAE or MAE speech communities by examining the number of hip-hop artists that use the word. The number of speakers using a new word is an excellent predictor of the word's later entrenchment in a speech community [28]. In other words, a word's long-term fate should be dependent less on a few popular individuals than on the speech community as a whole. Similarly, if exposure and learning were taking place from a listener's speech community (as opposed to listening to hip-hop), we might also expect that the number of hip-hop artists using a word would be a better predictor of comprehension vocabulary than sheer popularity of artists using the word. If however artist popularity is a better predictor of familiarity with a word than the number of artists using the word, we might conclude that listening to hip-hop outside of one's preferred artists, i.e. less engaged listening through either the radio or friends, is contributing to participants' AAE comprehension vocabulary. Therefore, prior to including either of these independent variables in the full model, I tested which was the better predictor: the number of hip-hop artists using a word, or the aggregate popularity of artists using a given word. The number of artists using a word is the sum of the artists in the present data using a word; the popularity score is the sum of the number of participants who said they listened to artists in the data that use the word.

These two variables were highly correlated (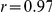). To determine which was the better predictor, I created new, independent predictors by first regressing overall artists onto artist popularity and using the residuals of this model as a predictor along with the original popularity score to predict AAE comprehension vocabulary. I then reversed the direction of the initial regression. When popularity score was the original predictor, the residualized number of artists was not significant. However, when the number of artists was the original predictor, the residualized popularity score was still significant. In other words, popularity score appears to be the better predictor of AAE comprehension vocabulary. This finding suggests that an effect of hip-hop artists outside the listener's preferred artists could be due to listening to hip-hop in somewhat less engaged contexts. I then added artists' aggregate popularity scores as a predictor to the above-specified model. Popularity score had a robust, positive association with comprehension vocabulary scores (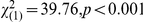).

**Figure 3:**
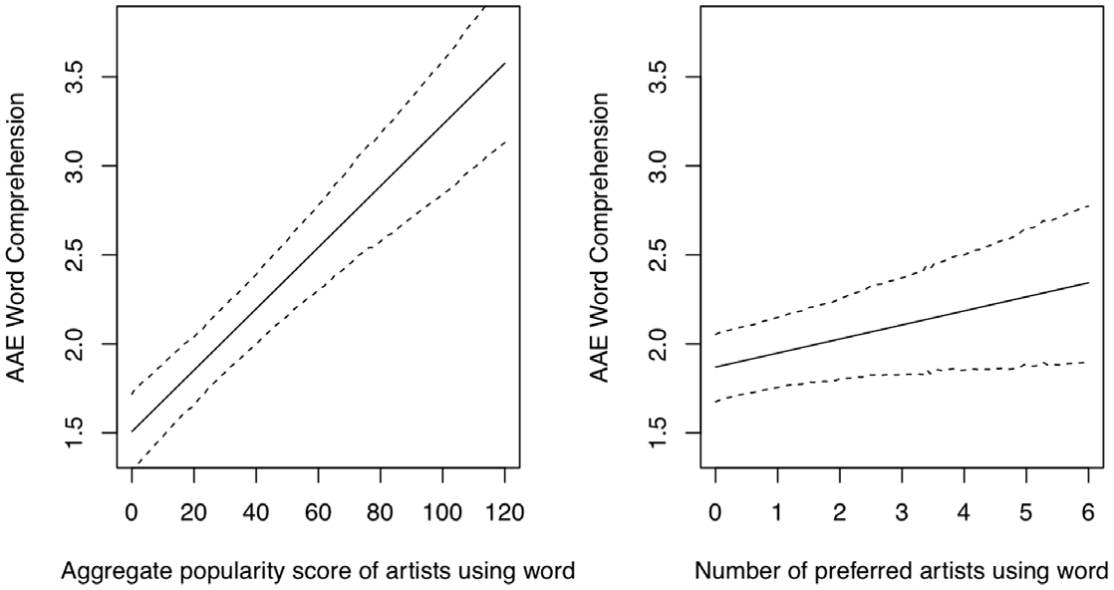
Partial effects of artists' aggregate popularity scores (left) and number of preferred artists (right) using a word on AAE vocabulary score. The dashed lines represent the 95% highest posterior density credible intervals.

As expected, the number of preferred artists using a word was correlated with the popularity score of artists using the word (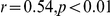). With this moderate level of correlation, any effect from the former could be due to the latter. When a new, independent predictor for preferred artists was created by using the residuals of a model in which preferred artists was regressed on popularity score, thus creating two independent variables, the number of preferred artists using a word was still significant (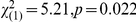). The partial effects of popularity score and preferred artists on AAE comprehension vocabulary are seen in Figure 3 and Table 2. For ease of interpretation, the figure gives the effect for the original preferred artists variable, which was very similar to that of the residualized preferred artists variable. Importantly, the direction of residualization reported here is the most stringent test for finding an effect of preferred artists. Results are given for a model in which influential datapoints were removed from the data. See supporting information S1 for further information on models.

**Table 2:** Model coefficients - individual word vocabulary score for participants listening to hip-hop.

	Estimate	Std. Error	t value	Pr( 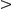 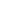 t 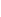 )
Intercept	1.0152	0.1629	6.2310	0.0000
Boondocks = yes	0.2221	0.1162	1.9107	0.0561
Mo'nique	0.1116	0.0387	2.8810	0.0040
Country	−0.0842	0.0332	−2.5362	0.0112
Hip-hop	0.0526	0.0238	2.2060	0.0274
Artists' Popularity	0.0172	0.0025	6.8538	0.0000
# Preferred Artists	0.0789	0.0348	2.2689	0.0233

N = 78. Dummy coding was used for the Boondocks variable, and the reference level is no knowledge of a *Boondocks* character.

This analysis revealed that use of a particular AAE word by a participant's preferred hip-hop artists was positively associated with the participant's knowledge of that word. This effect remained significant when the number of hip-hop artists listed by participants and the popularity scores of artists using a given word were included as predictors.

## Discussion

These results support the hypothesis that non-African-American young adults learn African-American English (AAE) vocabulary through listening to hip-hop. A positive association was observed between AAE comprehension vocabulary and the number of hip-hop artists participants listened to. Additionally, use of a particular word by a participant's preferred artists was predictive of the participant's knowledge of that word.

In addition, the present results suggest that vocabulary acquisition is a multifaceted process in which personal identity, in the form of cultural knowledge, social ties, and other musical elements, plays a crucial role. First, knowledge of African-American popular-culture figures was also associated with increased AAE comprehension vocabulary. Furthermore, the number of weak ties to African-Americans is a better predictor of AAE vocabulary knowledge than the number of strong ties, a finding which replicates the work on the strength of weak ties in the diffusion of information [29]. Third, a preference for country music was negatively associated with AAE comprehension vocabulary, yet this predictor was not negatively correlated with a preference for hip-hop. This finding is in line with country music's connection to cultural Whiteness in the United States [30], [31] (p. 24–30). Crucially then, this personal identity appears to be self-constructed. Factors that speakers have some degree of freedom in choosing, such as social networks and cultural consumption preferences, were predictive of AAE comprehension vocabulary. In contrast, no structural factors influencing identity, such as hometown population size, had a significant effect. Future work could further examine this distinction by looking at a more ethnically diverse non-African-American population, for example.

When accounting for these other possible means of vocabulary acquisition, hip-hop listening preferences are highly associated with AAE comprehension vocabulary. Still, the variables probed in this study are not meant to be exhaustively representative of speakers' media exposure and social networks. Rather, they are indicative of the types of factors that could affect AAE comprehension vocabulary and can suggest future directions for controlled laboratory studies. For example, in a comprehensive attempt to find interactions between all effects (see supporting information S1), none were found, which suggests that hip-hop listening preferences might have an effect on AAE comprehension vocabulary that is independent of listeners' social networks.

Nevertheless, for four reasons, the data in the current study suggest that AAE vocabulary acquisition will likely be linked to hip-hop listening preferences if other variables such as peer group usage and media exposure were able to be exhaustively examined and controlled for. First, some participants made explicit references to hip-hop lyrics in which a particular stimulus item occurred when defining the word. An example is the definition “where you go when you die/from the Bone Thugz [sic] song” [“Crossroads”, Bone Thugs-n-Harmony, 1996] for the stimulus item *crossroads* (“heaven”); for the item *make it rain* (“throw money up in the air”) one participant simply gave “Lil Wayne” as a definition. Lil Wayne, a well-known hip-hop artist, was featured in the song “Make it Rain” by Fat Joe, a less popular artist. Second, the correlations between hip-hop listening preferences and all other social and cultural factors such as social networks and non-musical media influences were low; the highest was 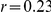. The lack of interactions found between predictor variables suggests that hip-hop listening preferences do exhibit an independent effect on AAE comprehension vocabulary. Third, the stimuli in this study were selected because of their relatively low rates of diffusion in Minnesota at the time participants took the study (Fall 2010). In fact, fully 76% of responses to the stimuli indicated that participants were not familiar with the word. This lower rate of diffusion, throughout both social networks and various forms of media, goes hand in hand with a decreased probability of multiple sources of exposure to a word. Finally, the fact that artists' aggregate popularity was a better predictor than the number of artists using the word suggests that even less involved listening to hip-hop could be responsible for familiarity outside of listeners' preferred artists. Listeners can often hear a popular artist such as Lil Wayne on the radio and through their friends, but may not actively choose to listen to this artist.

Most work on vocabulary acquisition from the media examines infant or non-native speaker populations, often in a pedagogical setting. If explicit attention is directed to the media, second-language learners are able acquire vocabulary from that source [32]. However, infants do not learn vocabulary from baby videos well [33]. Neither of these types of studies examine vocabulary acquisition as a result of speakers' voluntary interactions with various media forms. Adolescents can use media in constructing their personal identities [3], and learning in this context is most likely very different from pedagogical learning. For one, learner motivation is different. Adolescents could learn AAE vocabulary (from hip-hop) to be “cool”, to show affiliation with a social group, and to establish themselves as linguistic early adopters. Second-language classroom learning involves motivation to master the material and get a good grade.

Other work on vocabulary acquisition from the media makes claims that appeal to common sense. Reference [4] observes that buzzwords are clearly propagated by the media and are integrated into the active and passive vocabularies of at least some speakers. Examples of this phenomenon in the U.S. include trendy memes such as *truthiness*, Stephen Colbert's 2005 coining. But the words examined here are not buzzwords or memes; rather, they stem from a non-mainstream variety of English. Whereas buzzwords generally have a clear instance of coining, the same is not necessarily true for AAE vocabulary. And without an empirical study also examining social networks, it is impossible to tell whether this vocabulary is learned directly from the media for all speakers, or if they pass first from the media to influential speakers and then to other speakers. Previous linguistic theories posit that the media only influences a small number of speakers, who then pass on linguistic innovations to the majority of speakers in a community [19] (p. 356–357). In contrast, in this study, 48% of non-African-American participants listened to at least one hip-hop artist. Coupling this information with the present finding that young adults can learn AAE vocabulary from listening to hip-hop, as well as previous findings that adolescents use media as a form a self-socialization [3], it is possible that a larger percentage of the population than previously thought could be directly influenced from linguistic input from the media. In sum, further studies are needed on first-language vocabulary acquisition from voluntary interactions with various media forms.

Recent work shows an association between Scottish speakers who watch the London-based TV series *East Enders* and the use of phonetic features of Southern English [34]. Similarly, since speakers' comprehension vocabularies appear to be influenced by hip-hop, it is possible that speakers will subsequently *use* AAE vocabulary they hear in hip-hop songs. Since administering this survey, I have heard and seen members of the demographic studied here use *finna* (“gonna”), *chedda* (“money”), and *ballin'* (“living the good life”), three of the stimulus items of the present study. AAE is not the prestigious linguistic variety throughout the U.S. and is perceived as indicative of lower socio-economic status than MAE [35] (p. 223). However, its *covert prestige* [36] in the AAE and MAE speech communities also means its non-mainstream nature is perceived as cool [37]. This could make MAE speakers more motivated not only to learn, but also to use, AAE vocabulary they hear when listening to hip-hop.

For musical, linguistic, and social reasons, hip-hop represents a particularly interesting medium through which vocabulary acquisition can occur. The language of hip-hop lyrics is poetic, emotionally charged, accompanied by music and a fast tempo, and perhaps from another variety of English than the listener's. For these reasons, and because hip-hop artists rarely include lyrics in their liner notes, proper comprehension and transcription of hip-hop lyrics is “excruciatingly difficult” [9]. Thus it is far from established that hip-hop lyrics are understandable, and that unfamiliar words are presented in informative contexts therein. To have an idea of the degree of difficulty of learning vocabulary from hip-hop lyrics, the reader is encouraged to listen to Jay-Z and Kanye West's “Otis” (2011). A stimulus item in this study is featured somewhere between 0∶43–0∶57:

1. First, only listen to the song (http://www.youtube.com/watch?v=gMNVQeDN2q4);
2. Next, watch the video (http://www.youtube.com/watch?v=BoEKWtgJQAU);
3. Then, read the lyrics (http://www.killerhip-hop.com/jay-z-otis-lyrics-kanye-west/, first Jay-Z verse).

The reader can note at what point, if at all, he or she understands the meaning of the stimulus item. When compared to other examples, in my estimation this item in this context is easy to moderately difficult to understand.

The social factors accompanying hip-hop make for an even more intriguing medium through which vocabulary is learned. AAE is a socially stigmatized variety of English, traditionally spoken by members of an economically disadvantaged ethnic group. Yet African-Americans have a rich musical tradition (blues, jazz, gospel, soul, R&B, hip-hop) that mainstream America is keen to embrace [38]. This disparity has been called into question by African-Americans such as Roger Guenveur Smith, who has asked, “Why does everyone love Black music but nobody loves Black people? ” [38] (p. 5). Hip-hop is the current dominant form of African-American music, and the de-facto language of hip-hop is AAE. Thus hip-hop represents a prime medium through which speakers of MAE are exposed to AAE; as a result they associate the latter with coolness.

Paradoxically, as soon as speakers of MAE learn and adopt AAE vocabulary, hip-hop artists turn away from it. The word *bling-bling*, or simply *bling*, has hip-hop origins and was added to the Oxford English Dictionary in 2003, and to the Merriam Webster Dictionary in 2006 (see http://news.bbc.co.uk/2/hi/uk_news/magazine/3192258.stmh and http://www.mtv.com/news/articles/1564621/crunk-makes-it-into-dictionary.jhtml, respectively). Yet hip-hop artists no longer use this term. In 2004, MTV ran a cartoon showing the diffusion of *bling* starting with hip-hop artists and being adopted by subsequently more mainstream speakers, ending with a shot of *bling*'s lifespan, 1997–2003 (http://www.youtube.com/watch?v=JcMHC-W_Qmc). Street “cred”, the respect gained from familiarity with a tough urban lifestyle, is an essential part of a hip-hop artist's persona. Part of establishing street cred is using cutting-edge, in-group vocabulary not used by the middle-aged European-American woman at the end of the MTV clip. As a result, hip-hop artists are not motivated to make their lyrics accessible to their listeners. This is perhaps one of the reasons why hip-hop artists rarely include lyrics in their album liner notes.

The construction of a speaker's vocabulary as an adolescent and as an adult seems a vital component of his or her identity. Proper use and knowledge of slang, learned or technical terms, regional colloquialisms, and vocabulary from diverse languages and ethnic groups establishes a speaker as a credible member of a social group. Vocabulary use that a social group considers improper could result in mocking (friendly or unfriendly), ostracism, or even lawsuits and termination of employment (as an example, see http://articles.philly.com/2011-01-05/news/27010905_1_n-word-staff-meeting-federal-courts). Although crucial factors of a person's identity are developed during adolescence and young-adulthood [3], little research has been done on the myriad ways in which first-language vocabulary is acquired after puberty. Examining vocabulary acquisition through media sources such as hip-hop seems a promising first step in this line of research.

## Materials and Methods

### Ethics statement

The Institutional Review Board at the University of Minnesota approved this human participants research. Written informed consent was obtained from all participants. The IRB also approved the public availability online of information about artists in the survey as given below. IRB approval was also granted for the public availability of the full dataset.

### Participants, materials, design

One hundred sixty-eight undergraduate students in introductory undergraduate courses in music, linguistics, and sociology at the University of Minnesota participated in this online survey for course credit. One participant was African-American, and another gave an ethnicity of “bi-racial” without specifying the two ethnicies. Therefore, data from these participants were excluded so as to lessen the likelihood that participants could be familiar with the stimulus items from their everyday lives. The stimuli, available in Table 3, consisted of sixty-four vocabulary items, a subset of vocabulary specific to AAE. These were obtained from at least one of three sources: a native speaker of AAE; a dictionary of AAE [39]; or Rap Dictionary (http://www.rapdict.org/), an online source of AAE and other words used by hip-hop artists.

**Table 3:** Stimuli and their targeted AAE definitions.

Word	Definition	Word	Definition
*5*–*0*	police	*ghostride*	dancing while driving
*A-town*	Atlanta	*good hair*	straight hair
*CP time*	colored people's time	*gouda*	money
*The Nation*	Nation of Islam	*grip*	a lot
*(to be) ghost*	to leave/be out of here	*grip grain*	to have wood grain on your steering wheel
*ashy*	dry skin for African Americans	*guap*	lots of money
*ay yo trip*	check this out	*hard*	tough
*ballin*'	to live the good life/play basketball	*heezy*	variant of off the hook; cool, fun
*beezy*	woman	*hella*	very
*bones*	dollars	*humming*	bad odor/breath
*boo*	boyfriend/girlfriend/someone you love	*krump*	style of street dance
*boughie*	bourgeois	*mail*	money
*break someone out*	to tell on	*make it rain*	throw down money on people
*catch the vapors*	to get caught up in somebody else's affairs	*off the hook*	very good/new/crazy wild
*chedda*	cash	*player hater*	a person who's resentful/jealous of promiscuous people
*cheese*	money	*plex*	beef, as in issue
*chitlins*	chitterlings - pig intestines	*road dog*	friend
*cop my steezy*	copy someone's style	*roll deep*	in large numbers
*crossroads*	heaven	*rollie*	Rolex [watch]
*cuddie*	good friend	*saditty*	cocky
*dap*	greeting with hands like handshake	*skrilla*	money
*dead presidents*	cash; dollar bills	*straight cash*	only cash
*dollar cab*	subway	*stupid*	very; cool
*domino*	hundred dollar bill	*sweatbox*	small club
*duckets*	cash/money	*talking jazz*	say bad things about
*dukey rope*	fat gold chain	*toe up*	tore up; disheveled
*dun*	PERFECTIVE ASPECT	*trife*	troublesome/trifling
*face gator*	friend	*trill*	true/real
*facheezie*	for sure	*twurk*	dance
*feel me*	understand me	*what it do*	How are you?
*fetti*	money	*what it is?*	How are you?/What's up
*finna*	gonna	*wile out*	to flip out

### Procedure

Participants were first given the vocabulary items, presented one at a time, to freely define; they were told beforehand that they were participating in a survey on AAE and then were asked, “If you heard this word in a slang context, what would it mean?” Presentation order was randomized separately for each participant. Next, participants were asked their sex and age as well as free-response questions addressing their ethnicity, hometown (city/state or foreign country), musical tastes, social networks, and popular culture knowledge in that order. The two questions about musical preferences were (question labels in brackets):

1. What are your favorite genres of music? [Genres]
2. Name some of your favorite groups/artists in each genre (up to 5 per genre given above). [Artists]

Two questions about participants' social networks elicited ethnic information about participants' strong and weak ties:

1. Please list the first names of all the people you know that you would ask to help you move, along with their ethnicity (up to 15). [Move]
2. Please list the first names of any African-American friends/acquaintances you might have that you interact with on a weekly basis, either online or in person (up to 15). [Weekly]

Asking for first names of participants' strong and weak ties helps participants clearly enumerate friends, as opposed to asking for an imprecise estimate [40], [41]. The Move question elicits participants' strong ties, assuming that most people would not feel comfortable asking acquaintances to help them move. The Weekly question relaxes the criteria for association, thereby eliciting weaker ties.

Finally, participants responded to five popular-culture questions. These questions were selected for their diverse domains (given below in parentheses) as well as for the differing degrees of prominence of their subject matter in popular culture. All questions also incorporate varying degrees of AAE:

(music) Is Jay-Z married, and if so, to whom? [Jay-Z]
(sports, TV) What network is Charles Barkley a commentator for? [Barkley]
(TV) Please name a character from *The Boondocks*. [Boondocks]
(TV, movies) Who is Mo'nique? [Mo'nique]
(music) Please list as many song titles by Justin Bieber as you can (up to 5). [Bieber]

The responses to all of these questions were then used as predictor variables in a multiple linear regression analysis with participants' AAE vocabulary scores, described below, as the dependent measure.

### Data processing

The dependent measure, AAE comprehension vocabulary, was transformed to a scale of 1 to 5 from free-response form (see also [42] for a similar binning scheme). A value of 1 indicated no knowledge of the word; 76% of responses were given this value. A 5 was given if the definition corresponded to any attested meaning specific to AAE. Another 15.8% of responses were given values of 5. Responses indicating partial knowledge (8.2%) were given values of 2, 3, or 4. With 64 items, a participant's summed vocabulary score could range from 64 to 320.

Two researchers independently coded the definitions given for all 64 words; the Krippendorff's alpha coefficient [43] for inter-rater reliability was 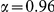. The corresponding figure for the responses that were not simply “not sure” (53.7% of responses) was 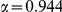. The researchers then met to examine definitions they did not classify identically. Genuine disagreements were resolved such that the dependent measure reflects rater agreement.

The 2009 estimates for a participant's City, County, and county's African-American (CountyAA) populations were recorded using U.S. census data. The census data are available at http://www.census.gov/popest/cities/files/SUB-EST2009-ALL.csv). Together, these population variables serve as rough estimates of the number of unknown speakers participants could potentially have interacted with on a regular basis, as well as casual exposure to AAE.

Responses to the first four popular culture questions were transformed from free-response form to a scale of 1 to 5. Again, 1s indicated no knowledge of the answer, while 5s indicated complete knowledge. Answers indicating partial knowledge were given values of 2, 3, or 4. A binary predictor variable of knowledge/no knowledge was determined to better fit the Jay-Z and Boondocks variables. For the Bieber question, responses were 1 plus the number of songs correctly identified. Inter-rater reliability values for responses to these questions were all 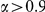.

If a social tie listed had two ethnicities, that person was counted as both ethnicities. I grouped social ties listed for the Move question into six ethnicities: African-American, Asian/Asian-American, European-American (White), Hispanic, Native American, and South Asian/Middle-Eastern/African. This last ethnicity is not standard but was used due to data sparsity. Mean values for each variable were used when no ethnicity data was given (n = 11 or 12 for Move questions and n = 3 for Weekly).

All artists listed were coded into one of nine possible genres, a subset of those in [44]: alternative, classical, country, folk, international, pop, rock, vocal/jazz/showtunes/oldies and hip-hop, the primary genre of interest. Participants were assigned numeric values for each genre corresponding to the number of artists they listed in that genre. Hence there were nine music-related predictor variables. Responses to the Genres question were used to disambiguate between two or more artists with the same name but different genres.

In total, 867 unique artists were listed, with The Beatles being the most popular (listed by 25/168 total participants).

### Availability of data

The entirety of the data discussed in this paper is available online at http://purl.umn.edu/116327. This includes the dataset off which the models were created for all 166+2 participants 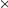
64 vocabulary items, the genre classifications for each of the 867 artists in the survey, and the number of times each artist was listed. No data potentially identifying participants are included, and the University of Minnesota IRB granted approval for the public availability of this dataset.

## Supporting Information

Supporting Information S1 **Details on statistical models and further data specifications.**
(PDF)
