# Supplementary material for: You Know What It Is: Learning Words through Listening to Hip-Hop
Source: PLoS One. 2011 Dec 21;6(12):e28248. doi: 10.1371/journal.pone.0028248 (PMC3244393; doi:10.1371/journal.pone.0028248)
Supplement: Supporting Information S1 — Details on statistical models and further data specifications. (PDF) [file pone.0028248.s001.pdf]

You know what it is:
Learning words through listening to hip-hop

Paula Chesley[1*]
1 Department of Linguistics, University of Alberta, Edmonton, Alberta, Canada
∗ **E-mail: pchesley@ualberta.ca**

Supporting information

Statistical analyses

The following section details the process by which statistical models were selected. See the subsequent sections for a discussion of models described in the main text.

Model selection

At the beginning of this research, the 26 predictor variables given in Table 1 were potentially predictive of the dependent measure. Of course, not all these variables would turn out to be significant predictors. Principal component analysis and factor analysis of the predictors are one way to reduce this number, but each is problematic. For example, in both of these methods, there is no relationship to the dependent measure.

Demographic	Social network	Musical preferences	Pop-culture
Sex (C)	MoveAA	Classical	Jay-Z (C)
Age	MoveAsian	Country	Barkley
City	MoveWhite	Folk	Boondocks (C)
County	MoveHispanic	Alternative	Mo'nique
CountyAA	MoveNative	International	Bieber
	MoveSAAME	Pop	
	Weekly	Rock	
		Vocal	
		Hip-hop	

Table 1. Possible predictor variables for predicting AAE vocabulary knowledge. (C) = categorical variable; all other variables are numeric. SAAME = South Asian/African/Middle Eastern.

To ensure that the findings were robust, four different methods were used:

1. A non-parametric dimension reduction technique (sliced inverse regression; see [1], [2]) to determine the predictors necessary for a linear model,
2. A non-parametric random forest model [3]. This model makes no assumptions about the distributions of the dependent or predictor variables,
3. A linear mixed-effects regression (LMER) model, with dependent measure as individual word score and random effects for Participant and Word,
4. A linear model with dependent measure as an individual's summed vocabulary score.

The model reported in the main text is the linear model in (4) above, with predictors selected from the dimension reduction technique in (1) as well as a stepwise backward elimination using the Akaike

Information Criterion [4] to eliminate superfluous predictors. This model is given in Table 1 in the main text.

A dimension reduction analysis such as sliced inverse regression can indicate the number of linear combinations of the predictor variables necessary to adequately describe the dependent measure. For the present data, one linear combination sufficed; a visual inspection determined that this dimension was linear. Therefore, interaction and quadratic terms of predictor variables were not needed. The number of predictor variables was then reduced with the use of a stepwise backward elimination method for dimension reduction objects [5]. Significant predictors retained in the model were Hip-hop, Country, MoveAA, Barkley, Boondocks, and Mo'nique; Jay-Z was also retained despite lack of significance ($\chi^2_{(1)} = 2.43, p = 0.121$).

Results from the random forest model, with conditional permutations to eliminate effects due to collinearity of predictors [6], revealed that the predictors Hip-hop, Country, Weekly, Barkley, Boondocks, Jay-Z, and Mo'nique are contributing to improved predictions for AAE comprehension score.

Finally, the LMER model in (3) was created using fixed effects yielded from steps (1) and (2) above. In this model, the predictor variables Hip-hop, Country, Weekly, Barkley, Boondocks, and Mo'nique were all significant at $p < 0.05$. Crossed random effects of Participant and Word were also included.

Model

The model reported in Table 1 of the main text was chosen for its interpretability as well as for its generalizability, agreeing with the LMER model as well as with the random forest and stepwise dimension reduction analyses. In this model, estimates correspond to β values, with the dependent measure being participants' overall AAE vocabulary scores (range: 64-320). All variables except for Boondocks are linear; Boondocks is a categorical predictor with treatment (dummy) coding. The reference level for the binary Boondocks predictor is no knowledge of a *Boondocks* character.

Residuals from the model given in Table 1 of the main text were normally distributed, barring a few outlier datapoints. To check for undue influence of outliers, datapoints with standardized residuals > 2.5 were removed and the model was re-fit to the data. All fixed and random effects were still significant predictors, with the same direction and similar magnitudes, and residuals were normally distributed. Influential datapoints were also examined and were determined not to be unduly affecting the model (for a discussion of influential datapoints, see [7], section 6.2.3).

The effect of preferred artists

The linear mixed-effects regression model given in Table 2 of the main text was fit to the data in the subsequent analysis on the influence of lyrics by participants' preferred hip-hop artists. Estimates in the model are β values, with the dependent measure as vocabulary score for individual words (range: 1-5). Again, the reference level for the binary Boondocks predictor is no knowledge of a *Boondocks* character.

This model included the crossed random effects of Participant and Word, seen in Table 2. Including these random effects in models allows for higher precision modeling of systematic variation and reduces the chance of spurious fixed-effects findings [8]. The main effect of Trial (position of the item in the experimental list) was not significant, nor did this variable vary by participant.

In this analysis, one participant and one word (*hard*) were determined to be unduly influencing the model. The participant's AAE comprehension vocabulary score was exceptionally high at over 3 standard deviations from the mean. The word *hard* had by far the lowest random effect of any word. Thus the model reported excludes data from this word and this participant. When these datapoints are included, the model is largely the same, except the effect of preferred artists decreases slightly (it is marginally significant; $\chi^2_{(1)} = 3.10, p = 0.078$). Residuals in this model were fairly normally distributed. Again, to check for undue influence of outliers, datapoints with standardized residuals > 2.5 (146; 2.85% of datapoints) were removed and the model was re-fit to the data with no significant changes.

Effect	SD	LR Statistic	p-value
Word	0.608	791.29	< 0.001
Participant	0.400	277.93	< 0.001

Table 2. Random effects for analysis including preferred artists. The likelihood ratio (LR) statistics and corresponding p-values were obtained by comparing the models with and without the given random effect substructure.

As presented, the model is as stringent as possible in detecting an effect of preferred artists. The residualization method used was regression of preferred artists onto popularity score, but one could also make the case for the opposite residualization. Such a residualization approach would regress artists' popularity scores onto the preferred artists and use the residuals for popularity score in addition to a model with the number of preferred artists. This is an intuitively appealing option, as the preferred artists variable includes motivated linguistic input, while the residualized popularity score variable could correspond to less motivated listening of hip-hop. In this analysis, the effect of preferred artists is highly significant ($\chi^2_{(1)} = 44.55, p < 0.001$), as is the residualized popularity score ($\chi^2_{(1)} = 36.16, p < 0.001$). As mentioned in the main text, if the preferred artists variable is used and the popularity score variable is excluded, the effect of preferred artists is similarly highly significant. Finally, a subsequent random forest analysis confirms the importance of preferred artists in predicting AAE vocabulary knowledge.

Further data specifications

Table 3 summarizes participants' self-reported ethnicities. Data from the participant who gave her ethnicity as bi-racial without specifying ethnicities were excluded as were data from the African-American participant, as was discussed in the main text. However, analyses that included these participants yielded the same results. Due to data sparsity, other participants who gave their ethnicites as bi-racial were given their non-White ethnicities as opposed to a new ethnicity.

Ethnicity	Number
African-American	1
Asian/Asian-American	17
Bi-racial (Asian/White)	2
Bi-racial (South-Asian [Indian]/White)	1
Bi-racial (ethniticies not given)	1
White (of European descent)	135
East-African	5
Hispanic	3
Native American	1
South Asian (Indian)	1
Not reported	1
Total	168

Table 3. Summary of self-reported participant ethnicities.

One hundred seventeen female and 51 male students enrolled in undergraduate classes participated in the study. Participant ages ranged from 16 to 48 (mean: 20.0, median: 19). Ninety per cent of participants were aged 22 or under.

Some participants did not provide American city and state information, either because they were from foreign countries, or because their response was underspecified. Foreign-born participants were given hometown values of 0 for City, County, and CountyAA; these values measure the number of potential (African-)American speakers with which these participants come into contact in their hometown. Treatments for these and other anomalous responses are given in Table 4.

Information given	# participants	Data treatment
Foreign country	5	City, County, and CountyAA populations given values of 0
"Puerto Rico"	1	City and County given mean population values of Puerto Rican *municipios*; CountyAA population given value of 0
"USA"	2	mean sample values for City, County, and CountyAA populations for non-foreign participants assigned
"Small town in the middle of Iowa"	1	City, County, and CountyAA values given mean values of City, County, and CountyAA populations for Iowa, respectively
"suburbs"	1	mean values for City, County, and CountyAA populations for the Minneapolis-St. Paul metro area assigned
"California"	1	Mean values for City, County, and CountyAA populations for California assigned

Table 4. Treatment for data with anomalous hometown information (either foreign country/underspecified American city/state information). US census data does not divide Puerto Rico into first-order administrative divisions such as cities; a *municipio* is equivalent to a county. Because of this, city and county information for this participant are the same.

Non-African-American rappers can use language differently than African-American rappers [9]. To ensure that the artists classified as hip-hop artists were actually likely to use AAE, all non-African-American hip-hop artists such as Eminem were classified as pop artists. However, when these artists were classified as hip-hop artists and the models were refitted to the data, the results were extremely similar to those reported. A few artists that are more likely to be classified as R&B artists, such as Beyoncé, were classified as hip-hop artists so that use of AAE could be gauged in a coherent music variable.

The effect of preferred artists

The method described of obtaining information about the number of artists using a particular word has two potential sources of noise. First, it is possible that lyrics on the Urban Lyrics information are incorrect. To minimize this possibility, known spelling variants of words were also queried. Second, the lyrics could be incomplete: users contribute to lyrics websites at their leisure. However, there is currently no gold standard of hip-hop lyrics: even *The Anthology of Rap* [10] appears to have many erroneous lyrics [11]. Additionally, the predictor variable measures use by particular artists and not frequency counts. It is much less likely that an error would be made for a given artist than for frequency of use by

that artist.

A second model was fit to the data using the number of songs in which the word is used (as opposed to number of artists) both from a participant's preferred artists and from artists' aggregate popularity scores. In this model, the number of overall songs the word was used in was a significant predictor, but the number of songs from the preferred artists was not significant. This mitigated effect could stem from the possibility that participants only listen to a few songs by a given artist as opposed to the entire repertoire.

Rater guidelines

For the dependent measure of word knowledge, no specific criteria were given for each word; instead, the general guidelines as described in the data processing section of the main text were given to raters. Raters then met to discuss each definition given, and the dependent measure reflects inter-rater agreement.

For the five popular culture questions, more specific criteria were available to readers for responses for each question. These criteria are given below; for responses other than those given, raters assigned the score they felt appropriate.

- **Jay-Z**. Participants should get a 1 if they say no, to someone other than Beyoncé, or NS (not sure). They get a 5 if they say anything like "Beyoncé" or "yes, to Beyoncé". They get a 2 if they say "yes" but don't say to whom, and they get a 4 if they correctly say Beyoncé but express some doubt, like a question mark or "to Beyoncé I think".

- **Barkley**. Participants get a 1 if they say NS or BET. They get a 2 if they say something vaguely basketball-related but not more, and they get a 3 or 4 if they say any sports-related TV station like ESPN or The Golf Channel. They get a 2 for TBS as that too is a Turner network but not the right one. Finally they get a 5 if they say TNT.

- **Boondocks**. Participants get 1 if they can't name any character and 5 if they can correctly name a character. If you see something marginal, such as information about the show without naming a character, feel free to give it a 2,3, or 4.

- **Mo'nique**. Participants get a 1 for NS and a 2 for "singer" as she technically did do some singing. Mo'nique was a TV show host and a comedian before starring in *Precious*, winning an Oscar for her role. If they list one of the following they get a 3: TV show host, actress, comedian. If they are list one of these but are unsure, give them a 2. If they list two of these, give them a 4. And if they list all 3 or can give details about any of these, as in "she was in Precious and she won an Oscar" then give them a 5. The more detailed info they can give the better.

- **Bieber**. Participants get a score of 1 for NS and one additional point for each Bieber song they can name. Song titles participants list that are almost right but not exactly (not only partially right) can get half a point.

Excluded participants

This was an online study, and some of the vocabulary items used as stimuli were unlikely to be known or used by participants. As such, it was possible for participants to look up the definitions of these words online at sites such as `www.urbandictionary.com`. However, throughout the recruitment process it was stressed that there are no right or wrong answers, and that what mattered was participants' own responses. Additionally, it was faster for participants to simply say they did not know the vocabulary item than to look it up online. The highest vocabulary score by participant was 249/320 (77.8% of the total points), and the median was 109 (34% of the total points). Still, researchers assessed that two of the

total 170 participants had provided definitions so close to those online that they must have been looking up definitions. These participants' data were removed from the analyses.

References

1. Li KC (1991) Sliced Inverse Regression for Dimension Reduction. Journal of the American Statistical Association 86: 316-327.

2. Weisberg S (2002) Dimension Reduction Regression in R. Journal of Statistical Software 7: 1-22.

3. Breiman L (2001) Random Forests. Machine Learning 45: 5-32.

4. Akaike H (1974) A new look at the statistical model identification. IEEE Transactions on Automatic Control 19: 716-723.

5. Cook R (2004) Testing predictor contributions in sufficient dimension reduction. Annals of Statistics 32: 1062-1092.

6. Strobl C, Hothorn T, Zeileis A (2009) Party On! a New, Conditional Variable Importance Measure for Random Forests Available in the `party` Package. Technical Report 50, Institut für Statistik, Ludwig-Maximilians-Universität München.

7. Baayen RH (2008) Analyzing Linguistic Data: A practical introduction to statistics using R. Cambridge, U.K.: Cambridge University Press. 368 p.

8. Baayen RH, Davidson DJ, Bates D (2008) Mixed-effects modeling with crossed random effects for subjects and items. Journal of Memory and Language 59: 390-412.

9. Cutler C (2007) The Co-Construction of Whiteness in an MC Battle. Pragmatics 17: 9-22.

10. Bradley A, DuBois A (2010) The Anthology of Rap. New Haven, CT: Yale University Press.

11. Sanneh K (2010) Word: Jay-Z's "Decoded" and the language of hip-hop. The New Yorker December 6. Available: `http://www.newyorker.com/arts/critics/atlarge/2010/12/06/101206crat_atlarge_sanneh`, accessed 8 October 2011.
